# Clinical Efficacy of Sodium Butyrate in Managing Pediatric Inflammatory Bowel Disease

**DOI:** 10.3390/life15060902

**Published:** 2025-05-31

**Authors:** Adrian Goldiș, Radu Dragomir, Marina Adriana Mercioni, Diana Sirca, Christian Goldiș, Ileana Enatescu, Laura Olariu, Oana Belei

**Affiliations:** 1 Department of Gastroenterology and Hepatology, “Victor Babeș” University of Medicine and Pharmacy, 300041 Timișoara, Romania; goldis.eugen@umft.ro; 2 Department of Obstetrics and Gynecology, “Victor Babeș” University of Medicine and Pharmacy, 300041 Timișoara, Romania; 3 Faculty of Medicine, “Victor Babeș” University of Medicine and Pharmacy, 300041 Timișoara, Romania; diana-cristiana.sirca@student.umft.ro (D.S.); christian.goldis@student.umft.ro (C.G.); 4 Applied Electronics Department, Faculty of Electronics, Telecommunications and Information Technologies, Politehnica University Timișoara, 300223 Timișoara, Romania; 5 Twelfth Department, Neonatology Clinic, “Victor Babeș” University of Medicine and Pharmacy, 300041 Timișoara, Romania; enatescu.ileana@umft.ro; 6 First Pediatric Clinic, “Victor Babeș” University of Medicine and Pharmacy, 300041 Timișoara, Romania; olariu.laura@umft.ro (L.O.); belei.oana@umft.ro (O.B.); 7 First Pediatric Clinic, Disturbances of Growth and Development on Children Research Center, “Victor Babeș” University of Medicine and Pharmacy, 300041 Timișoara, Romania

**Keywords:** Crohn’s disease, ulcerative colitis, sodium butyrate, inflammatory bowel disease, CRP, fecal calprotectin, children

## Abstract

Background: Few studies have evaluated the efficacy of butyric acid in treating children with inflammatory bowel disease (IBD). In children and adolescents with recently diagnosed IBD, the purpose of this research was to assess the efficacy of oral sodium butyrate (the product-patented, sustained and targeted-release form of butyrate MSB^®^) as an adjunct to conventional treatment. Methods: This trial was unicentric, prospective, randomized, and placebo-controlled. An amount of 150 mg sodium butyrate once a day (Group A), or a placebo (Group B) were randomly assigned to patients with ulcerative colitis or Crohn’s disease, aged 7–18 years, who were receiving conventional medication based on the severity of their conditions. Disease activity, C-reactive protein (CRP), and fecal calprotectin concentration differences between the two study groups at 12 weeks of the trial were the main outcomes. Results: With 44 patients in Group A and 44 in Group B, 88 individuals with initially active illness finished the research. Most patients experienced remission by week 12 of the study (36 patients in Group A with sodium butyrate, 81.82%; 21 patients in Group B with placebo, 47.73%). Between the two groups, a significant difference in disease activity was seen (*p* < 0.001). The sodium butyrate group appeared to have less systemic inflammation than the other group, as evidenced by the significantly lower CRP levels in Group A (18.14 ± 11.19 mg/L) compared to Group B (57.00 ± 33.28 mg/L) at 12 weeks (T2) (*p* < 0.001). No negative effects were recorded by any of the patients. Fecal calprotectin in Group A dropped much more after 12 weeks (T2) (*p* < 0.001), suggesting that the sodium butyrate group was better able to regulate intestinal inflammation. Conclusions: In newly diagnosed children and adolescents with IBD, a 12-week sodium butyrate supplementation did demonstrate effectiveness as an additional treatment.

## 1. Introduction

Inflammatory bowel disease (IBD) is a cumulation of heterogeneous complex immune disorders affecting the gastrointestinal (GI) tract and can be divided into two major phenotypes: Crohn’s disease (CD) and ulcerative colitis (UC), both of which share a common physiopathological substrate—the presence of an active inflammatory process in the gut.

In addition to having similar symptoms like fatigue, diarrhea, and abdominal pain, Crohn’s disease and ulcerative colitis (UC) are both chronic inflammatory bowel diseases (IBD) that are characterized by immune-mediated inflammation of the gastrointestinal tract and overlapping risk factors like genetics and environmental triggers [1].

IBD often leads to complications that significantly impact a patient’s quality of life, including bone issues, thrombosis, and extraintestinal manifestations. Over the past two decades, there has been a decrease in colectomy rates for UC, with earlier procedures being performed. Factors influencing colectomy include male sex, pancolitis diagnosis, younger age, and extraintestinal manifestations presence [2,3,4,5,6].

Regarding pediatric IBD (pIBD), global rates are rising rapidly, particularly due to the rising incidence of CD, with statistics showing 25% of all IBD subjects are under the age of 18, and approximately one quarter of pIBD patients are under the age of 10 [7]. Most recent studies show a rapid increase in very early onset IBD (VEO-IBD), defined as diagnosis before the age of 6 years [8]. Patients diagnosed with IBD during childhood present unique challenges, often showing more severe intestinal involvement and faster progression. Regular follow-ups with pediatric gastroenterologist are essential to monitor growth, delayed puberty, nutritional deficiencies, treatment side effects, infections, extraintestinal manifestations and, last but not least, psychosocial support, since 25–40% of adolescent IBD patients show signs of clinical depression, affecting school attendance, medication adherence and overall QoL [9,10].

The Paris Classification of inflammatory bowel disease is used to classify pIBD and takes into consideration the age of diagnosis, disease location, behavior and the presence or absence of growth delay [11]. Consequently, pIBD treatment is tailored based on disease severity and its primary goal is to achieve clinical and histological remission, while minimizing medication side effects. As of now, treatment strategies include amino-salicylates (AS) and antibiotics (ATB) for mild to moderate cases, while steroids (S) are used for more severe cases. Immunomodulators like thiopurines (T) help maintain remission, while biologics such as anti-TNF agents (TNF) are reserved for refractory high-risk cases. Nutritional support, including enteral nutrition, is also a key component in promoting growth and managing disease symptoms [12].

The location, severity, and systemic effect of pediatric inflammatory bowel disease (IBD) vary from those of adult-onset IBD because of developmental considerations. Children frequently exhibit more widespread bowel involvement, including ileocolonic Crohn’s disease and pancolitis in ulcerative colitis (73.1% in children compared to 30.2% in adults), as well as greater rates of transmural inflammation and strictures. These variations are explained by increased immunological dysregulation throughout development phases and genetic predisposition associated with early-onset illnesses. Growth impairment (such as stunted height or delayed puberty) and psychological stresses associated with body image and adolescence are among the particular clinical problems associated with pediatric IBD. Aggressive treatments, such as anti-TNF medicines, are necessary to prevent long-term damage, although intestinal inflammation frequently endures even after symptoms go away, requiring endoscopic surveillance. The necessity for organized transition programs is further highlighted by the fact that moving from pediatric to adult treatment increases the risk of relapse since it disrupts care continuity [13,14,15,16,17].

A pivotal factor in the pathogenesis and progression of IBD, in both adult and pediatric populations, is the microbiome. The human gut microbiota is the new actor on the stage of discoveries regarding immune-modulated diseases—whether we are talking about gastrointestinal diseases, allergies, neurodegenerative diseases or even cancer. As stated from previous research, the dynamics of the gut microbiome in patients with IBD differ from those of healthy controls, characterized by a newly defined healthy plane (HP). The intestinal microbiome composition of IBD subjects periodically intersected the HP, mostly during remission phases, but also frequently deviated from it, especially in patients who had surgical resections or a more aggressive treatment plan, due to intensified flares of the disease [18]. Dysbiosis has been defined as increased levels of aggressive bacterial species (such as *Akkermansia* and *Bacteroides fragillis*) and decreased protective species (such as *Faecalibacterium prausnitzii*, *Roseburia intestinalis* and *Eubacterium*) and is considered a key factor in the etiology of IBD [19,20,21].

One of the beneficial components of the intestinal microbiota, butyrate-producing bacteria have been nominated as “The Sentinel of the Gut”, given their well-documented beneficial effect on the GI tract [21]. Butyrate is a short-chain fatty acid (SCFA) produced in the intestinal lumen through dietary fiber fermentation and, alongside other SCFAs such as acetate and propionate, serves as the primary energy source for the colonocytes and strengthens the intestinal barrier [22]. Among the beneficial roles of sodium butyrate (the product-patented, sustained and targeted-release form of butyrate MSB^®^) in the GI tract counts the modulation of local immunity, by stimulating anti-inflammatory processes and inhibiting the production of proinflammatory cytokines, regulating macrophages, dendritic cells and T cells [23].

Numerous studies assessed sodium butyrate’s potential role in alleviating IBD symptoms, reducing flares and prolonging remission. In experimental studies, oral supplementation with sodium butyrate was found to inhibit the NF-κB inflammatory signaling pathway, function as an HDAC inhibitor and modulate gene expression, and most importantly, protect intestinal health in animal models, alleviating chronic colitis and acute DSS-induced colitis [24].

This study aims to evaluate the effectiveness of oral administration of sodium butyrate as an addition to standard therapy in children and adolescents diagnosed with inflammatory bowel disease at onset. The primary objective was to assess the difference in disease activity and fecal calprotectin measured at enrolment and after 12 weeks of sodium butyrate administration associated with standard basic therapy. If proven effective, butyrate could represent a safe and accessible adjunct to current therapy regimens and improve long-term prognosis in childhood IBD.

## 2. Materials and Methods

### 2.1. Study Design and Population

**Figure 1:**
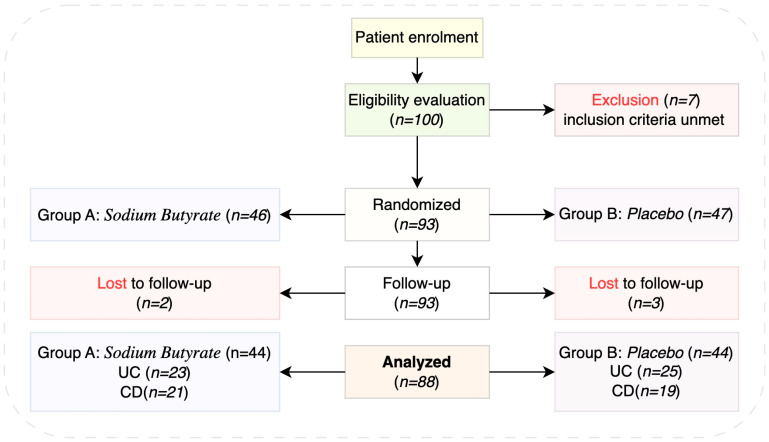
Pediatric IBD patients’ enrolment algorithm.

**Figure 2:**
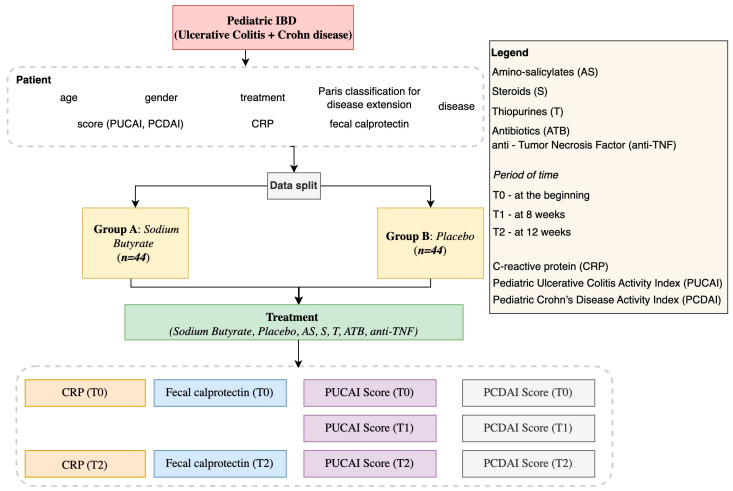
Pediatric IBD study design.

For children and adolescents with inflammatory bowel disease (IBD) at the outset of the illness, a single-center, prospective, randomized, placebo-controlled trial was carried out to assess the efficacy of oral sodium butyrate as an adjuvant treatment (Figure 1). Comparing sodium butyrate, a short-chain fatty acid with anti-inflammatory qualities, to conventional medication alone, the study sought to determine whether adding it may enhance clinical results. A variety of clinical measures, such as inflammatory markers (C-reactive protein—CRP, fecal calprotectin), and Pediatric Ulcerative Colitis Activity Index (PUCAI) and Pediatric Crohn’s Disease Activity Index (PCDAI) scores, were tracked throughout a predetermined time period after participants were randomly allocated to receive sodium butyrate or a placebo (Figure 2).

### 2.2. Inclusion Criteria

- Age 7–18 years
- Newly diagnosed inflammatory bowel disease
- Signed informed consent
- No probiotics or other dietary supplements were taken in the last 2 weeks before enrolment in the study.

The study population comprised 88 patients diagnosed with IBD who met the inclusion criteria. Patients were stratified into two distinct groups based on a computer-generated randomization that was used to allocate patients into one of two groups.

*Group A* (44 patients, 50%, 23 patients UC, 21 patients CD) was given standard treatment in accordance with the current national standards, along with 150 mg of microencapsulated sodium butyrate for 12 weeks.

*Group B* (44 patients, 50%, 25 patients UC, 19 patients CD) was the group that got the standard treatment in accordance with the current national standards, along with a 12-week *placebo*.

### 2.3. Endpoints

The interest endpoints of the study were:

• Relapse patient percentage: The proportion of patients who experienced a relapse: determining the effectiveness of treatment for pediatric children in both groups was a key goal, even if it differed between the two groups.

CRP response: Evaluated at baseline of the patient enrolment and after 12 weeks of treatment.

• Fecal calprotectin: Evaluated at baseline of the patient enrolment and after 12 weeks of treatment.

• Pediatric Ulcerative Colitis Activity Index (PUCAI) and Pediatric Crohn’s Disease Activity Index (PCDAI) scores: Evaluated at baseline of the patient enrolment and after 8 and 12 weeks of treatment, respectively.

### 2.4. Statistical Analysis

The statistical analysis of this study aimed to evaluate the effectiveness of different types of treatment among pediatric patients with IBD. To accomplish this, rigorous statistical methods tailored to the analyzed data were applied.

To compare groups for continuous variables such age, PUCAI, PCDAI scores, fecal calprotectin, and CRP, an Independent Samples T-test was used. The findings were reported using *p*-values to evaluate statistical significance and were presented as mean ± standard deviation (SD). Using the Chi-square test, relationships were examined for categorical factors including gender, disease, Paris classification for disease extension, disease severity and treatment regimens (e.g., the use of amino-salicylates, steroids, thiopurines, antibiotics, or anti-TNF).

Changes in PUCAI, PCDAI scores (Table 1), CRP, and fecal calprotectin were analyzed across multiple time points (PUCAI, PCDAI at baseline and after 8 and 12 weeks of treatment, and CRP, fecal calprotectin at baseline and after 12 weeks of treatment). These analyses primarily relied on an Independent Samples T-Test and a Paired Samples T-test. Results were reported in their respective units, such as CRP (normal reference range < 5.0 mg/L) and fecal calprotectin (normal reference range < 50 µg/g) (Table 2), with a threshold for statistical significance set at *p* < 0.05.

Finally, the study conducted additional analyses to evaluate the predictive value of CRP and fecal calprotectin thresholds. ROC curve analysis was used to determine sensitivity, specificity, and the area under the curve (AUC) for baseline CRP and fecal calprotectin as prognostic markers. Correlation analyses further explored the relationship between PUCAI, PCDAI scores and clinical outcomes, including treatment response, CRP and fecal calprotectin.

## 3. Results

The comparison of baseline characteristics between patients in Group A and Group B revealed no statistically significant differences across demographic and clinical parameters (Table 3). Age, gender distribution, and statistical significance (*p*-values) are the parameters compared between Group A (Sodium Butyrate, N = 44) and Group B (Placebo, N = 44) in the table. There is not a significant difference in the mean age between the two groups (11.6 ± 3.5 years vs. 11.9 ± 3.5 years, *p* = 0.630). There are no notable variations in these baseline characteristics, indicating a well-balanced study group. The gender distribution is likewise consistent, with 50% of the participants in Group A being female and 52.27% in Group B (*p* = 0.831). The PCDAI and PUCAI scores for Group A (Sodium Butyrate) and Group B (Placebo) are compared in the table, as is the distribution of Crohn’s disease and ulcerative colitis. With a *p*-value of 0.669, which indicates no statistically significant difference, the percentage of patients with Crohn’s disease (47.73% vs. 43.18%) and ulcerative colitis (52.27% vs. 56.82%) is comparable across groups. Confirming that both groups are well-matched in terms of disease type and severity at baseline, the distribution of PCDAI and PUCAI scores also exhibits the same pattern. The Paris classification for illness extension in Groups A and B is also shown in the table. While L2 and L3 denote the areas of Crohn’s disease, E1–E4 correlate to varying degrees of ulcerative colitis. There are no statistically significant variations between the two groups’ patient distributions across these categories (*p* = 0.904 for all categories). This implies that the two groups’ baseline illness extents were well matched, guaranteeing comparability about the location and severity of the disease prior to therapy. Combinations of amino-salicylates (AS), steroids (S), thiopurines (T), antibiotics (ATB), and anti-TNF medication are among the treatment plans utilized in Group A and Group B that are contrasted in the table. There are no statistically significant variations in the distribution of treatment plans between the two groups (*p* = 0.954 for all categories). As a result, any effects seen in the research are less likely to be impacted by variations in previous medication usage. This shows that both groups had similar baseline treatment exposures. The disease severity (based on PUCAI and PCDAI scores variation from baseline and after 12 weeks of treatment) in Groups A and B is shown to have a statistically significant *p*-value of 0.001, indicating that a considerably greater percentage of patients in the sodium butyrate group (81.82%) were in remission than those in the placebo group (47.73%). Interestingly, only the sodium butyrate group experienced severe illness (2.27%), although the placebo group saw intermediate disease more frequently (36.36% vs. 6.82%). These variations imply that, in comparison to the placebo group, a slightly higher percentage of patients in the sodium butyrate group (four patients, 9.09%) had more severe illness after 12 weeks of treatment.

The evidence that is now available indicates that age does affect therapy response in pediatric IBD, however it is conflicting and sometimes complicated by research design problems. Growing older seems to be a predictor of a worse clinical response to therapy with exclusive enteral nutrition (EEN) induction for Crohn’s disease, especially in pediatric populations. Additionally, some research suggests that younger children may react better to specific biologics. For example, one study found that vedolizumab only had an exposure-response relationship in pediatric patients with Crohn’s disease who weighed less than 30 kg at week 6, indicating that younger or smaller children might handle it better. Children often exhibit greater remission rates across a variety of medications when comparing pediatric to adult populations more generally. These include corticosteroids (prolonged response 50–61% versus 32–44% in adults), thiopurines (85% vs 31% at 6 months), and infliximab (56% vs 28% at 1 year). However, as pediatric studies usually assess patients with shorter illness duration and more aggressive “top-down” treatment approaches soon after diagnosis, experts warn that these apparent age-related variations may be mostly caused by study design issues rather than age itself. However, children’s continuously greater response rates add confidence to the advantages of early, intensive treatment approaches for pediatric IBD [27,28].

Table 4 presents the count of patients diagnosed with Crohn’s disease and Ulcerative Colitis, categorized by severity and remission status, within two groups (A and B).

The dataset consists of 88 pediatric patients, 40 (45%) of whom have been diagnosed with Crohn’s disease and 48 (55%) with ulcerative colitis. At group-level data analysis, Group A comprises 44 patients, of which 36 (81.82%) are in remission (16 CD, 20 UC), 1 patient (CD) is categorized as mild, and 7 patients (16%) are in the Moderate to Severe category (Moderate: 2 CD, 1 UC; Severe: 2 CD and 2 UC). Comparably, Group B contains 44 patients, 21 of whom (47.73%) are in remission; nevertheless, there are more Mild cases (6 patients, 13.64%) and Moderate to Severe cases (17 patients, 38.63%).

Group B includes a slightly greater percentage of active cases (23 in Group B vs. 7 in Group A) and a smaller percentage of patients in remission (21 vs. 36 in Group A). Group A had more Ulcerative Colitis (25 vs. 23 in Group B), but Group A has a greater prevalence of Crohn’s disease (21 vs. 19 in Group B). Greater remission rates in Group A might be a sign of improved treatment response or illness management. A greater percentage of Moderate and Severe cases are seen in Group B, indicating the need for more intensive care. The notably increased percentage of Moderate patients in Group B compared to Group A may have an impact on the results comparison, as baseline illness severity is a crucial factor impacting treatment response and outcomes. Group B shows more patients in the Moderate group; their overall prognosis, symptom improvement, or remission rates may look less favorable due to the beginning severity, rather than variations in treatment efficacy.

Table 5 shows the differences in inflammatory markers (CRP and fecal calprotectin) between Group A and Group B at baseline (T0), and 12 weeks later (T2). The C-Reactive Protein (CRP) levels in Group A (88.66 ± 37.43 mg/L) and Group B (83.93 ± 40.32 mg/L) were comparable at baseline (T0) (*p* = 0.570, not significant). CRP levels, however, were considerably lower in Group A (18.14 ± 11.19 mg/L) than in Group B (57.00 ± 33.28 mg/L) after 12 weeks (T2) (*p* < 0.001), suggesting that the sodium butyrate group had reduced systemic inflammation more than the other group. In terms of fecal calprotectin (FC), Groups A and B had comparable FC levels at baseline (T0) (937.27 ± 713.86 µg/g) and 845.25 ± 608.46 µg/g) (*p* = 0.517, not significant). When compared to Group B (311.73 ± 248.69 µg/g), Group A’s fecal calprotectin decreased by a considerably larger amount after 12 weeks (T2) (*p* < 0.001), indicating that the sodium butyrate group had better control over gut inflammation.

**Figure 3:**
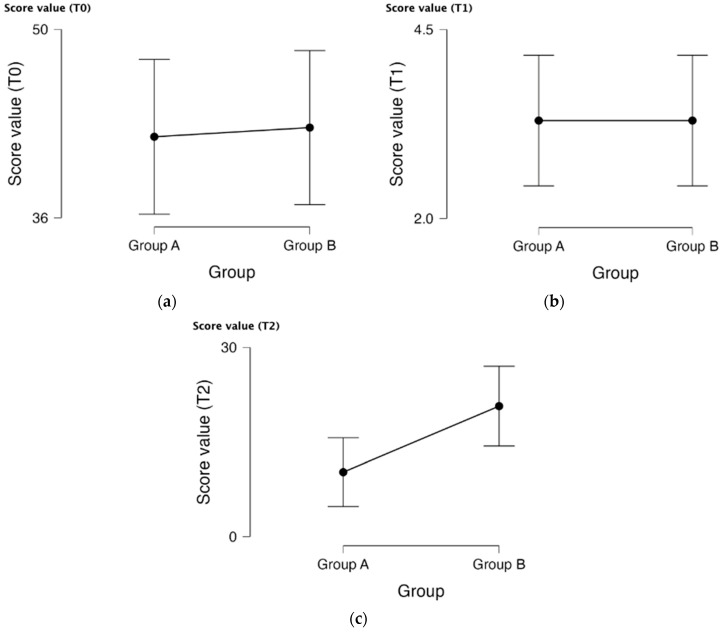
Descriptive plots. (**a**) Score value based on PUCAI and PCDAI variation from baseline (T0) for Group A and Group B; (**b**) Score value based on PUCAI and PCDAI variation after 8 weeks (T1) for Group A and Group B; (**c**) Score value based on PUCAI after 12 weeks of treatment (T2) for Group A and Group B.

Figure 3 shows the change in score values for two groups, Group A and Group B, over time (at baseline T0, after 8 weeks T1, and after 12 weeks T2). These score values are based on PUCAI and PCDAI, which are indicators of disease activity and severity. Plots of the mean scores for each group at each time point are displayed, and variability is represented by error bars. Baseline (T0) displays the score values at the beginning of the trial for both groups. Although Group A’s mean score is slightly lower than Group B’s, the overlap between their error bars raises the possibility that the difference is not statistically significant. (T1) displays the score values after 8 weeks of therapy. Group A and Group B’s mean score values seem to be very comparable. The large overlap of the error bars indicates that there is now no noticeable distinction between the groups. Both groups have significantly improved, as seen by the significantly lower score values on the y-axis compared to T0. The score values are displayed after 12 weeks of therapy (T2). The mean score for Group B is greater than that of Group A. The first two time points already show a large error bar without statistical significance, thus the third time point also has no meaningful significance.

**Figure 4:**
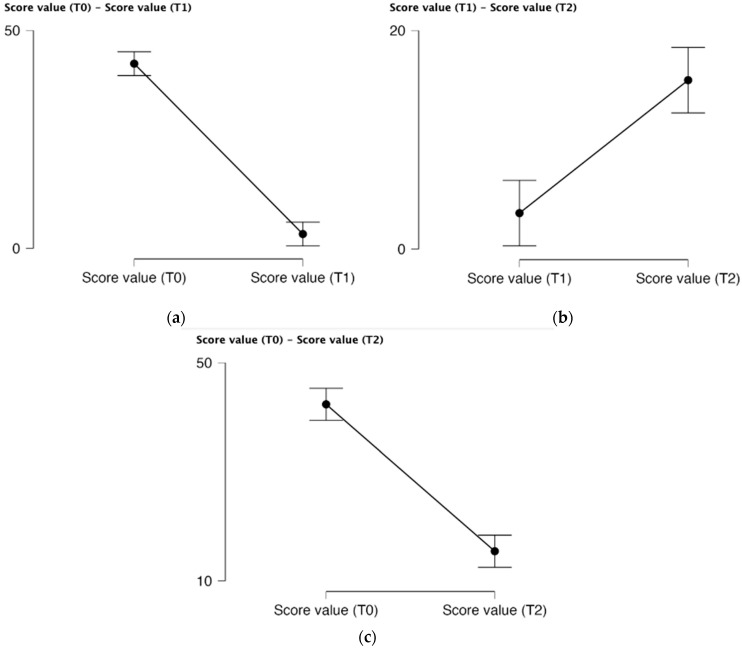
Descriptive plots applying Paired Samples T-test for score value at baseline, after 8 weeks and after 12 weeks. (**a**) Score value based on PUCAI and PCDAI variation from baseline (T0) and after 8 weeks of treatment (T1); (**b**) Score value based on PUCAI and PCDAI variation after 8 weeks (T1) and after 12 weeks of treatment (T2); (**c**) Score value based on PUCAI and PCDAI variation from baseline (T0) and after 12 weeks of treatment (T2).

Applying Paired Samples T-test for score value at baseline, after 8 weeks and after 12 weeks, the Figure 4 shows how the PUCAI and PCDAI scores for Group A and Group B changed over the course of treatment. Score value based on PUCAI and PCDAI variation from baseline (T0) and after 8 weeks of treatment (T1) displays the overlapping error bars indicate that although Group A’s mean score is slightly lower than Group B’s, this difference likely is not statistically significant. In terms of disease activity, this suggests that the groups were rather well-matched at the beginning of the trial. The mean scores for Group A and Group B are quite comparable, with significant overlap in the error bars, according to the score value based on PUCAI and PCDAI fluctuation after 8 weeks (T1) and after 12 weeks of treatment (T2). This implies that the groups’ responses to treatment at this time point do not differ significantly based on PUCAI and PCDAI. Group B had a much higher mean PUCAI score than Group A, and the error bars appear to overlap less than at the previous time periods, according to the score value based on the PUCAI and PCDAI change from baseline (T0) and after 12 weeks of therapy (T2). This raises the possibility of different results. Group A seems to exhibit less disease activity (more cases in remission, inactive 36, and just 8 active disease cases) than Group B (21 patients in remission and 23 active disease cases) at 12 weeks.

**Figure 5:**
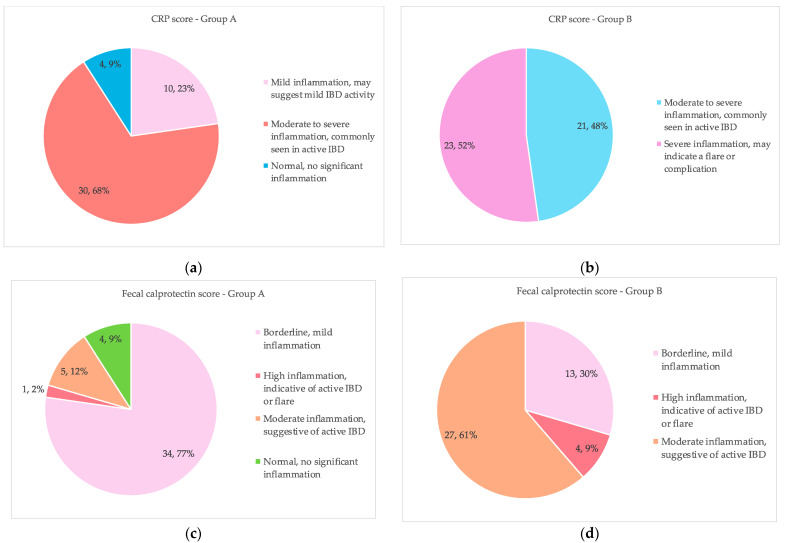
CRP and fecal calprotectin inflammatory markers analysis after 12 weeks of treatment in each group (**a**) CRP score in Group A; (**b**) CRP score in Group B; (**c**) Fecal calprotectin score in Group A; (**d**) Fecal calprotectin score in Group B.

Using CRP and fecal calprotectin as indicators, Figure 5 shows the levels of inflammation in two groups of IBD patients following 12 weeks of therapy. According to subplot (a), 68% (30) of patients in Group A have moderate to severe inflammation, 23% (10) have mild inflammation, and 9% (4 patients) have no noticeable inflammation, according to CRP values. According to subplot (b), 52% (23) of patients in Group B had severe inflammation, whereas 48% (21) have moderate to severe inflammation, according to CRP levels. 77% (34) of patients in Group A have borderline mild inflammation, 12% (5 patients) have severe inflammation, 9% (4) have moderate inflammation, and 2% (1) have no significant inflammation, according to subplot (c) of fecal calprotectin levels. According to fecal calprotectin levels, 61% (27) of patients in Group B have severe inflammation, 30% (13 patients) have borderline mild inflammation, and 9% (4) have moderate inflammation, as shown in subplot (d). By comparing the levels of these inflammatory markers in the two groups, this study aids in determining how well the medication reduces inflammation in IBD patients.

## 4. Discussion

According to the findings of our study of pediatric populations, *sodium butyrate* supplements were found to be a useful adjunctive therapy for recently diagnosed IBD in children and adolescents.

By restoring intestinal homeostasis and reducing inflammation, butyrate, a short-chain fatty acid generated by gut microbial fermentation, has several positive benefits on inflammatory bowel disease (IBD). By stimulating the production of important proteins like synaptopodin (SYNPO), which stabilizes the actin cytoskeleton and tight junctions, lowering mucosal permeability, and promoting tight junction assembly through the activation of signaling pathways like AMPK and Akt, it improves the integrity of the epithelial barrier. As a histone deacetylase (HDAC) inhibitor, butyrate also causes more histones to be acetylated, which alters gene expression by suppressing NF-κB activity, a key transcription factor that drives the production of proinflammatory cytokines, and upregulating anti-inflammatory genes. By restricting the migration, release of proinflammatory cytokines, and formation of neutrophil extracellular traps (NETs), butyrate reduces the activation of immune cells like neutrophils and the secretion of inflammatory mediators like IL-8. This epigenetic regulation helps to reduce mucosal inflammation. Butyrate also promotes the generation of mucus by epithelial cells and alters both innate and adaptive immunity, including the activation of regulatory T cells, which helps the gut’s immunological tolerance. Supported by in vitro research, animal models of colitis, and observations of decreased butyrate-producing bacteria and butyrate levels in IBD patients, butyrate’s combined effects on immune modulation, inflammation suppression, and epithelial barrier reinforcement underscore its therapeutic potential in IBD [29,30].

The outcomes of the few trials that have looked into oral sodium butyrate supplementation in adult IBD patients so far are mixed [31]. According to the research [32], restoring butyrate-producing bacteria seems to be essential for UC and CD patients to have long-term remission [33].

Following two months of oral administration of 1800 mg of sodium butyrate or a placebo daily in addition to standard therapy, this randomized, controlled trial [34] did not find any changes in calprotectin levels or IBD activity. However, patients with CD predominated in the Italian trial, in contrast to our sample, which was dominated by UC.

Similar to our study, 30 UC patients receiving fixed dosages of mesalazine were randomized in the Vernia et al. study [35]. One group was given a placebo for six weeks, while the other group was given 4 g of sodium butyrate. Following the intervention, 7/15 of the patients who received sodium butyrate experienced remission of their condition, and an additional 4/15 showed improvement, whereas 5/15 of the patients who received a placebo experienced remission and 5/15 showed improvement.

Di Sabatino et al. [36] conducted an observational study on 13 CD patients. Following 8 weeks of oral supplementation with 2 × 2 g sodium butyrate, patients who had CD saw a 53% (7/13) remission of their condition, and an additional 2/13 saw a decrease in disease activity. Patients with IBD can safely utilize sodium butyrate. No adverse events were recorded by any subjects during the research. It is important to highlight the great safety of sodium butyrate that has been documented in all published investigations, even those that used rectal administration and those that used large oral dosages of butyrate.

According to Lin et al., butyrate caused very little mucosal injury in the colon and distal ileum of neonatal rats when administering more than 150 mmol/L [37]. More human in vivo research is required to advance our present understanding of butyrate-mediated effects on colonic function in both health and illness, even though the majority of studies indicate that butyrate has positive benefits [38].

The possibility that successful butyrate supplementation could last far longer than the 12 weeks we employed in our trial cannot be ruled out, though. Because sodium butyrate is more beneficial in individuals who have lower disease activity or who are in remission, this might account for the disparate outcomes that different researchers have found.

New therapeutic alternatives for gastrointestinal problems are being treated medically by gastroenterologists and surgically by surgeons, respectively, thanks to the active formulations of exogenous sodium butyrate that are currently accessible and intended to be released within the colon [39].

The two conditions, UC, and CD, really have different inflammatory lesion locations in the gastrointestinal system, and patients are treated in different ways. This significant variation might help to explain why sodium butyrate works so well to treat inflammatory lesions in a mouse model’s intestines but not so well to treat IBD in people [40,41,42,43].

The authors of this study demonstrated that, regardless of genotype, the butyrate step-up therapy approach may be a successful treatment plan for all congenital chloride diarrhea (CLD) patients. This strategy can make it easier to keep surveillance on the patient week after week until the ideal dosage is achieved. This strategy could not be contraindicated in the case of a first failed butyrate’s therapeutic course. Patients with CLD may be able to safely and permanently reduce the intensity of their diarrhea with butyrate medication [44].

Although lower levels of butyrate and/or the microbes that produce this metabolite are linked to disease and worse health outcomes, butyrate has also demonstrated protective actions in the context of intestinal diseases like IBD, graft-versus-host disease of the gastrointestinal tract, and colon cancer [45].

In a few clinical trials, oral butyrate has demonstrated some promise as an adjuvant therapy, even for individuals who are resistant to treatment. Butyrate is a short-chain fatty acid that is important for the immunological homeostasis of the colonic mucosa. A patient with UC who was resistant to pharmacological and dietary therapies was described in this case study [46] as responding favorably to an oral butyrate trial.

Derivatives of butyric acid are also utilized as biologically active agents to treat a range of human medical disorders. Positive outcomes have been observed in the treatment of colorectal cancer, Helicobacter pylori infection eradication therapy, irritable bowel syndrome, inflammatory bowel disorders, and functional constipation. Furthermore, butyric acid and its derivatives have beneficial extraintestinal effects in the context of cerebral ischemia, insulin resistance, hypercholesterolemia, and hemoglobinopathy. The therapeutic benefits of butyrate in treating pediatric obesity were validated by a randomized clinical study [29,47,48,49,50,51,52,53,54].

The purpose of our pediatric IBD population study was to assess how well oral sodium butyrate affected disease activity. Our research’s relatively small study group (*n* = 88) is one of its drawbacks, although it is still among the biggest on IBD patients. Our objective was to acquire a clinical assessment and practical implementation of the obtained data, rather than evaluating the impact of sodium butyrate supplementation on the intestinal microbiota’s composition.

Despite encouraging preclinical and some clinical data showing its anti-inflammatory and barrier-enhancing properties, important questions about butyrate’s effectiveness, the best ways to deliver it, and its mechanisms in humans still need to be answered. For this reason, more research on butyrate in inflammatory bowel disease (IBD) is essential. For instance, whereas butyrogenic diets and oral butyrate supplements have demonstrated advantages in lowering inflammation and preserving remission, butyrate enemas have yielded inconsistent outcomes, and it is unclear how active inflammation affects butyrate metabolism and epithelial response. Butyrate oxidation and utilization in the gut epithelium may be impeded by inflammation, which might restrict its therapeutic efficacy during illness flares. Further research is also necessary to understand the intricate interactions with immune cells like neutrophils and the variation in butyrate-producing bacteria across patients. Therefore, well-designed clinical trials are required to determine if butyrate alone or in conjunction with other medications can successfully induce and sustain remission in IBD, optimize formulations and dosage, and determine which patient subgroups are most likely to show benefits [30,55].

## 5. Conclusions

All things considered, age-related variables and molecular processes such as sodium butyrate are essential for maximizing IBD treatment plans for children. A 12-week sodium butyrate supplementation did show promise as an extra therapy for newly diagnosed IBD in children and adolescents. When combined with standard therapy delivered in accordance with national regulations, the administration of sodium butyrate at a dosage of 150 mg/day significantly reduced the rate of relapse to 12 weeks in children with inflammatory bowel disease. Sodium butyrate is a well-tolerated product with no adverse effects that reduces inflammatory activity in children with Crohn’s disease and ulcerative colitis. The gastrointestinal tract and the overall system benefit greatly from butyric acid’s nutritive, immunomodulatory, and protective properties. Because they break down quickly at the point of manufacture or release, natural forms do not offer any further therapeutic potential. To precisely establish the dosage and time of administration to sustain long-term remission of inflammatory bowel disease, further research on bigger cohorts of pediatric patients is needed.

## Figures and Tables

**Table 1:** Classification of disease severity (activity).

	Score	Severity	Status
PUCAI *	0–10	Remission	Inactive
	10–34	Mild	Active
	35–64	Moderate	Active
	65–85	Severe	Active
PCDAI *	6.8 ± 6.6	Remission	Inactive
	18.7 ± 7.3	Mild	Active
	38.5 ± 12.9	Moderate	Active
	54.2 ± 14.0	Severe	Active

* Pediatric Ulcerative Colitis Activity Index (PUCAI) [25] and Pediatric Crohn’s Disease Activity Index (PCDAI) [26] scores.

**Table 2:** Inflammatory markers.

	Score	Severity
CRP * (mg/L)	<5.0	Normal, no significant inflammation
	5–10	Mild inflammation, may suggest mild IBD activity
	>10	Moderate to severe inflammation, commonly seen in active IBD
	>50	Severe inflammation, may indicate a flare or complication
Fecal calprotectin * (µg/g)	<50	Normal, no significant inflammation
	≤200	Borderline, mild inflammation
	≤500	Moderate inflammation, suggestive of active IBD
	>500	High inflammation, indicative of active IBD or flare

* Pediatric Ulcerative Colitis Activity Index (PUCAI) [25] and Pediatric Crohn’s Disease Activity Index (PCDAI) [26] scores.

**Table 3:** Demographic and clinical characteristics.

Characteristics	Group A Sodium Butyrate (N = 44)	Group B Placebo (N = 44)	*p* Value
**Age ^(a)^**	11.6 ± 3.5	11.9 ± 3.5	0.630
**Gender ^(b)^**			
**F**	22 (50%)	23 (52.27%)	0.831
**M**	22 (50%)	21 (47.73%)	0.831
**Disease ^(b)^**			
**Crohn disease**	21 (47.73%)	19 (43.18%)	0.669
**Ulcerative Colitis**	23 (52.27%)	25 (56.82%)	0.669
	**Score ^(b)^**		
**PCDAI**	21 (47.73%)	19 (43.18%)	0.669
**PUCAI**	23 (52.27%)	25 (56.82%)	0.669
**Paris classification for disease extension ^(b)^**			
**E1**	5 (11.36%)	4 (9.09%)	0.904
**E2**	4 (9.09%)	6 (13.64%)	0.904
**E3**	7 (15.91%)	5 (11.36%)	0.904
**E4**	7 (15.91%)	10 (22.73%)	0.904
**L2**	10 (22.73%)	10 (22.73%)	0.904
**L3**	11 (25.00%)	9 (20.45%)	0.904
**Regimens used ^(b)^**			
**AS**	4 (9.09%)	3 (6.82%)	0.954
**AS, S**	6 (13.64%)	5 (11.36%)	0.954
**AS, S, ATB**	2 (4.55%)	4 (9.09%)	0.954
**AS, S, T**	5 (11.36%)	6 (13.64%)	0.954
**AS, S, T, ATB**	4 (9.09%)	2 (4.54%)	0.954
**S, T**	11 (25.00%)	11 (25.00%)	0.954
**S, T, ATB**	6 (13.64%)	4 (9.09%)	0.954
**T, anti-TNF**	4 (9.09%)	6 (13.64%)	0.954
**T, anti-TNF, ATB**	2 (4.54%)	3 (6.82%)	0.954
**Disease severity ^(b)^**			
**Remission**	36 (81.82%)	21 (47.73%)	<0.001
**Mild**	1 (2.27%)	6 (13.64%)	<0.001
**Moderate**	3 (6.82%)	16 (36.36%)	<0.001
**Severe**	4 (9.09%)	1 (2.27%)	<0.001

^(a)^ Mean ± SD; ^(b)^ Percentage; Amino-salicylates (AS), Steroids (S), Thiopurines (T), Antibiotics (ATB), anti-Tumor Necrosis Factor (TNF).

**Table 4:** Disease severity by group.

Group	Severity	Crohn’s Disease	Ulcerative Colitis	Total
		**21**	**23**	**44**
**Group A**	Remission	16	20	36
	Mild	1	0	1
	Moderate	2	1	3
	Severe	2	2	4
		**19**	**25**	**44**
**Group B**	Remission	6	15	21
	Mild	4	2	6
	Moderate	8	8	16
	Severe	1	0	1
**Total**		**40**	**48**	**88**

**Table 5:** Inflammatory markers at baseline, after 8 weeks, and after 12 weeks of treatment.

Inflammatory Marker		Group A (*N* = 44)	Group B (*N* = 44)	*p*-Value
**CRP ^(a)^**	**Baseline** (T0)	88.66 ± 37.43	83.93 ± 40.32	0.570
	**After 12 weeks** (T2)	18.14 ± 11.19	57.00 ± 33.28	<0.001
**Fecal calprotectin ^(b)^**	**Baseline** (T0)	937.27 ± 713.86	845.25 ± 608.458	0.517
	**After 12 weeks** (T2)	125.80 ± 164.07	311.73 ± 248.69	<0.001

^(a)^ mg/L, ^(b)^ µg/g; only T0 and T2 were measured.

## Data Availability

The data presented in this study are available on request from the corresponding author. The data are not publicly available due to privacy and ethical restrictions.
